# Dislocation of the Femur on the Dorsum Ilii, Reducible without Pulleys, or Any Mechanical Powers: Three Cases

**Published:** 1852-03

**Authors:** W. W. Reid


### Part 3.—Selections.

### ARTICLE I.

*Dislocation of the Femur on the Dorsum Ilii, reducible without Pulleys, or any other Mechanical Power. Three Cases.* An Essay read before the Monroe County Medical Society, at its Annual Meeting, in the city of Rochester, on the 8th May, 1850. By. W. W. Reid, M. D.

[We extract the following cases from an exceedingly well written article under the above title, upon dislocations of the hip joint, published in the Buffalo Medical Journal, for August, 1851.

The cases will sufficiently show the plan of reduction proposed by Dr. Reid, but we are sorry our limits forbid the publication of his remarks in full, as they are judicious and philosophical.]

*Case* 1.—In the spring of 1844—[I give this case from recollection, the notes which I made of it having been mislaid]—I was called to see a strong, robust Irish woman, of whom they gave me the following history: Four days previous, while out at washing about three-quarters of a mile from her own residence, she slipped and fell down a flight of steps—could not rise—and when helped up, could not stand. She made a great out-cry but as no blood was visible, she was thought to make a great "fuss for nothing." Her husband, who was an intemperate carman, was sent for. He put her on his cart, drove her home three-quarteas of a mile; when he arrived there, not being able to lift her, he dumped her down at the gate as he would a load of dirt. The neighboring women helped him carry her in, and placed her in bed. For four days they assiduously fomented her hip, of which she complained greatly; but it swelled considerably and became

"black and blue." They now began to think the woman was "*hurted*." In this condition I found her. A single glance at the position of the knee and toe, created a strong suspicion of dislocation, but an attempt to *abduct* and rotate the limb, gave great pain and determined the nature of the accident. Although the patient was suffering considerably, I was in extacies, and felt really obliged to her, not so much, I hope, for dislocating her hip, as for the opportunity she afforded me to reduce it. I called in Doctors M. Strong and the elder Bradley, and Mr. now Dr. Hammond, to assist me. I stated to them my determination to reduce it, if possible, without the use of pulleys, and explained my method. Nevertheless I had provided myself with compound pulleys, to be used in case of failure. As the accident was of four days standing, the hip considerably swollen and inflamed, and the patient quite muscular, I took the precaution to bleed her freely, and give her tart-antimony till nausea was produced. She was in the meantime placed on a lounge, on which a wide board was laid and covered with a folded quilt. This made a firm table about fourteen inches high, and about twenty inches wide, which gave me the opportunity of throwing the whole weight of my body on the flexed limb, if I wished, while it gave me perfect command and control over it in every way. The patient was placed on her back, and a sheet folded lengthwise thrown across the upper edges of the pelvis bones, and each end given to an assistant, for the purpose of fixing the pelvis. Placing myself on the right and injured side, I seized the knee with my left hand, and the ankle with my right; I then flexed the leg upon the thigh; at the same time, slowly carried the knee and dislocated femur, over the sound one, pressing it firmly down upon it—and upward over the pelvis, constantly pressing it close to the body, moving it upward with a circular sweep over the abdomen, till the thigh was in a line with the right side of the body and the knee, pointing towards the right axilla. While the thigh was being carried up to this position, the bone or axis of the femur, was performing a kind of rotation on itself, whereby the toe was coming more outward and the heel more inward. In other words, as the knee went upward, the *obturator externus*, *quadratus*, &c., drew the head of the bone downward, and inward towards its socket. When the knee and thigh were in the position above indicated, the heel was strongly rotated inward, the knee drawn outward and the foot carried across the thigh of the sound side, when the head slipped into its place, and the limb glided gently down into its natural position. In doing all this, comparatively very little force was employed, and very little pain produced, for the obvious reason, that, by this evolution, the muscles that were in a state of extreme tension and irritation by the displaced bone, were gradually relieved and relaxed, as the head of the bone descended and approximated its proper place, which it did by the action of the same extended muscles.

It will be perceived, that by this mode of operating, we make a *lever* of the shaft or bone of the femur, and a fulcrum of the edge of the pelvis—and by this means lift or dislodge the head of the bone,—while the abductor muscles draw it downward and inward, making it, as it were, *back into* its place, through the rent of the capsular ligament. Whereas, if it were drawn by direct force, as by the pulley, the head and neck of the bone would act as a kind of hook, and would tear away the capsular ligament, if it were only slit, and as I believe it often, if not always, does tear off the tendon of the *pyriformis*, as I shall endeavor to show presently; for the *abductor* muscles are so strained, and hold the head of the bone so firmly to the dorsum, behind the ridge of the *acetabulum*, that it is next to impossible for it to mount over this ridge and into the socket, and must therefore descend behind it tearing everything before it—ligaments, muscles and all—and hence the immense power required to reduce it by these means, and hence, too, the failures, the fractures of the neck, and the cripples, that have been made for life, by this barbarous and unscientific mode of reduction.

*Case* 2.—On the 31st of July, 1849. Mrs. Cornelius Christie, aged about 38 years, was thrown from the top of a load of household furniture, with a small child in her arms. Motherlike, she held fast to the child, which received no harm; but, falling among and upon the furniture, she had the perineum and vulva considerably larcerated, and her right hip dislocated. I saw her within an hour after the accident. Doctors Bowen, Brown, and Holton, were in attendance when I arrived in company with Dr. E. P. Langworthy. The patient was placed at once in the position as already described in case No. 1, when I proceeded, in like manner, to operate; but the wound in the perineum and vulva occasioning great pain, on the attempt to flex the thigh, I desisted, and gave a full dose of morphine—not having any chloroform on hand. We waited three-fourths of an hour for the effect of the morphine. I then, as already described, seized the knee with one hand—the ankle with the other—flexed the leg on the thigh—the thigh on the pelvis, carrying it *inward and over the sound limb*, then upward over the abdomen, till the thigh was nearly parallel with the right side—then rotated the heel inward, carried the foot over the sound thigh, and the knee outward, when by a gentle oscillation and rotation of the thigh, the head slipped into the socket. The whole time required in this operation did not exceed *two minutes*. The force employed, and the pain suffered, were too trifling to be named.

*Case* 3 —On the 2d of Dec., 1849, early in the morning, I met Dr. E. M. More, Prof. of Surgery in the Woodstock and Berkshire schools of medicine. He informed me he had been called up in the night to attend a case of dislocated hip. I jestingly said, "I wish you would let me show you how to reduce it." He replied as jocosely, "I understand you have got some new-fangled notions about dislocations, and I should like to see you try your skill." He desired me to explain my method. I did so, illustrating it by manipulations on the skeleton in his office. He agreed that I should make the attempt; but, that the full merit of my mode of operating should be brought out, he proposed that I should have no aid from any of the usual adjuvants, such as the warm bath, nauseating doses of antimony, bleeding, opium, nor chloroform. To all this I consented.

The patient, William Fagan, was a strong muscular Irishman, 52 years of age. He was placed on a lounge, on a board covered with a folded blanket,as already discribed---two assistants, one on each side, steadied the pelvis. I proceeded in all respects as above stated in the two preceding cases, and in about *two or three minutes* reduced the dislocation. Doctors More and Cruttenden, Mr. D. Bly, and other students of Dr. M. were present.

To those who have never witnessed this method of operating, these statements may seem incredible, yet so simple, easy and short is it, that Dr. More declared, that "hereafter any fool might reduce dislocation of the hip on the *dorsum ilii*." Although in the three cases given above, I used a low table, yet I believe the floor is better, and all that is necessary. I

used, too, a folded sheet thrown over the pelvis, and had it held down on each side by an assistant; but even this is unnecessary, and is, moreover, always in the way, after the thigh has been flexed to a right angle with the spine or axis of the body; when the thigh has reached this position we have perfect control of the pelvis, and can fix it firmly, by pressing the thigh strongly down upon it. So simple, too, is the operation, that if the patient be a female, and it were required to reduce the joint without exposing the person, it can be done, under a light covering, or under even her own dress, if sufficiently loosened.

On the 18th of December, just after the occurrence of the third case above narrated, Dr. More had a subject in process of dissection by his students, when he proposed to me that we dissect up the muscles of the hip joints, leaving them *in situ*; dislocate the bones, and then operate on them by traction in the usual way, and also by flection after my method, in order that we might observe the condition and action of the muscles, before and during both modes of operation. We found it impossible by the power of our hands alone to force the head of the bone through the capsular ligament, till we made a slight incision into it. The head then shot through it, tearing it sufficiently to permit its passage, but then the ligament seemed to fit close around the neck of the bone. As the head passed out backward and upward, it caught the tendon of the pyriformis, *tearing it off as it passed underneath and above it*, which, if it had remained entire, would have brought its tendon, like a cord, across the neck close to the head, lashing it firmly down to the dorsum of the ilium. We were at the time inclined to attribute its rupture rather to the decayed state of the subject, than to excessive distension by the dislocation. But precisely the same thing occurred in dislocating the other hip. It is true this muscle was also in the same *stale* state; and the accident may, perhaps, have happened in both instances from the like cause.

When dislocated, the head of the bone rested on the *gluteus minimus* muscle! The *gluteus medius* and *maximus* were shortened and relaxed—so also were the *iliacus internus*, *psoas magnus*, *adductor triceps* and *pectineus*. Till now I had supposed that this last named muscle would have been among those that were put upon the stretch. Posteriorly the *obturator internus*, *genelli* and *quadratus* were greatly strained; and it was apparent, that the *pyriformis*, if it had not been torn off, would have been even more so. Anteriorly, the *obturator exturnus* was stretched, seemingly, to its utmost, *adducting* the bone powerfully. It is this powerful muscle, which so firmly fixes the limb ; turns the toe and knee inward ; prevents rotation and abduction, and gives such excruciating pain to the patient when any such attempts are made.

Here, then, are two sets of muscles, acting in direct antagonism to each other, and both strained to their utmost tension. One set, drawing the bone backward and rotating it *outward*. The other, *abducting* and rotating it *inward*. Some might be inclined to puzzle themselves to know how these two set of muscles, one situated before and the other behind, could both be in a state of tension, when the bone is drawn backward toward and in the direction of the latter. The explanation is very easy. Although the head of the bone is thrown backward, yet the great *trochanter* and shaft of the bone is thrown forward and rotated inward. So that the *pyriformis*, *obturator internus*, &c., which are inserted at the root of the *trochanter*, are necessarily elongated, while the anterior *obturator externus* runs backward behind and around the bone, to be inserted at the root of the trochanter, in order to rotate the limb outward it must also be strained just in proportion as the limb is rolled inward, and the trochanter is carried upward. The *quadratus* is stretched for the same reason, viz: its point of insertion is carried upward and inward.

After having carefully noted the relative position of the bone and muscles, we made traction on the femur, downwards and inward, over the sound limb, as we are directed by the most approved authors, but the moment the attempt was was made, the muscles already named as being in a state of tension, became more tense, and bound the head of the bone more firmly down on the *dorsam ;* and although the muscles about the joint were separated from each other—were loose, without vitality and almost in a state of decomposition—yet it was with very great difficulty that we could bring the head of the bone down ; and when we did so, we carried away part of the capsular ligament, and if the *pyriformis* had not been already torn, it is very probable that it would have been torn now. But when we *abducted, flexed, and carried the limb up over the pelvis*, as has been stated, the reduction was effected with the utmost ease. We varied and repeated our experiments on both joints, as often as the subject would admit, and always with the same results. I was here enabled to correct one error which I had commited in operating. If we carried the knee above the *umbilicus*, and pressed the thigh down close to the body, on a line with the side, the knee pointing towards the axilla, as I had always done, we brought the great tendon of the *gluteus maximus* into strong tension, which would compress the great trochanter so hard, that it prevented the head from mounting over the edge of the acetabulum. The reduction was effected much easier by carrying the knee and thigh about as hig as the umbilicus, then abducting and rotating the thigh.

To Dr. More, who so kindly offered me the opportunity to demonstrate the correctness of both my theory and practice, I am much indebted and obliged.

From the foregoing facts and observations, gentlemen, I deduce the following propositions:

1. The chief impediment in the reduction of dislocations, is the indirect action of the muscles that are put upon the *stretch* by the mal-position of the dislocated bone, and not by the *contraction* of the muscles that are shortened.

2. That muscles are capable of so little extension, without hazard of rupture, beyond their moral strength, that no attempt should be made to stretch them further, in order to reduce a dislocation, if it can possibly be avoided.

3. The general rule for reducing all luxations should be, that the limb or bone should be carried, moved, flexed or drawn, in that direction which will relax the distended muscles.

4. Dislocation of the hip on the *dorsum ilii*, an accident so serious to the patient, and so formidable to all surgeons, is reduced with the greatest ease, in a few minutes, without much pain, without an assistant, without pulleys, without "Jarvis' Adjuster," or any other mechanical means, simply by flexing the leg upon the thigh, carrying the thigh over the sound one, upward over the pelvis, as high as the umbilicus, and then by *abducting* and rotating it.

